# Cognitive behaviour therapy for non-cardiac pain in the chest (COPIC): a multicentre randomized controlled trial with economic evaluation

**DOI:** 10.1186/s40359-015-0099-7

**Published:** 2015-11-24

**Authors:** Peter Tyrer, Helen Tyrer, Sylvia Cooper, Barbara Barrett, Stephanie Kings, Valentina Lazarevic, Kate Bransby-Adams, Katherine Whittamore, Gemma Walker, Antoinette McNulty, Emma Donaldson, Luke Midgley, Shani McCoy, Rachel Evered, Min Yang, Boliang Guo, Yvonne Lisseman-Stones, Asmae Doukani, Roger Mulder, Richard Morriss, Mike Crawford

**Affiliations:** Centre for Mental Health, Imperial College, Claybrook Road, London, W6 8LN UK; King’s Health Economics, King’s College, London, De Crespigny Park, London, SE5 8AF UK; East Midlands Clinical Research Network, Institute of Mental Health, Nottingham, NG7 2TU UK; North West London Clinical Research Network, Hammersmith Hospital, London, W12 0NN UK; Berkshire Healthcare NHS Foundation Trust, Skimped Hill Lane, Bracknell, Berkshire RG12 1BQ UK; Central and North West London NHS Foundation Trust, Hampstead Road, London, NW1 7QY UK; School of Public Health, Sichuan University, Chengdu, Sichuan China; Faculty of Medicine & Health Sciences, University of Nottingham, Queen’s Medical Centre, Nottingham, NG7 2UH UK; Kings Mill Hospital, Sutton-in-Ashfield, Nottinghamshire, NG17 4JL UK; London School of Hygiene and Tropical Health, Keppel Street, London, WC1E 7HT UK; Department of Psychological Medicine, University of Otago, Riccarton Avenue, PO Box 4345, Christchurch, 8140 New Zealand; Centre for Mental Health, Imperial College, 7th Floor, Commonwealth Building, Hammersmith Hospital, London, W12 0NN UK

**Keywords:** Cognitive behaviour therapy, Non-cardiac chest pain, Randomized trial

## Abstract

**Background:**

Most patients with chest pain have nothing wrong with their cardiac function. Psychological forms of treatment for this condition are more likely to be successful than others.

**Methods/design:**

A two-arm parallel controlled randomized trial of standard care versus a modified form of cognitive behaviour therapy for chest pain (CBT-CP) in patients who have attended emergency hospital services. Inclusion criteria include (i) emergency attendance more than once in the previous year with chest pain when no physical pathology has been found, (ii) aged between 16 and 75, (iii) signed consent to take part in the study. Exclusion criteria are (i) under current psychiatric care, (ii) those who have had new psychotropic drugs prescribed within the last two months, (iii) are receiving or about to receive a formal psychological treatment. Those satisfying these criteria will be randomized to 4–10 sessions of CBT-CP or to continue with standard care.

Participants are randomized using a remote web-based system using permuted stacked blocks stratified by study centre. Assessment is carried out at baseline by researchers subsequently masked to allocation and at 6 months and 1 year after randomization. The primary outcome is the Health Anxiety Inventory score at 6 months, and secondary outcomes are generalised anxiety and depressive symptoms, the Lucock Health Anxiety Questionnaire adapted for chest pain, visual analogue scales for chest pain and discomfort (Inskip Scale), the Schedule for Evaluating Persistent Symptoms (SEPS), health related quality of life, social functioning and medical resource usage. Intention to treat analyses will be carried out with clinical and functioning data, and a cost-utility analysis will compare differences in total costs and differences in quality of life using QALYs derived from the EQ-5D. The data will also be linked to another parallel study in New Zealand where 126 patients with the same inclusion criteria have been treated in a similar trial; the form of analysis of the combined data has yet to be determined.

**Discussion:**

The morbidity and costs of non-cardiac chest pain are substantial and if a simple psychological treatment given by health professionals working in medical departments is beneficial it should prove to be of great value. Combining data with a similar study in New Zealand is an additional asset.

**Trial registration:**

ISRCTN14711101 (registered 05/03/2015)

## Background

Chest pain is one of the most common reasons for attending an Accident & Emergency Department. This is understandable as cardiac conditions often present with chest pain and many sufferers require emergency interventions to save life. However, only a minority of patients presenting with chest pain have a demonstrated physical cause for their conditions. The experience of chest pain, even if not related to cardiac pathology, is often alarming and frightening, and if inadequately managed, tends to reinforce rather than resolve the problems.

Atypical, better termed non-cardiac, chest pain, can have a number of underlying pathologies. Most importantly, it can be an indicator of genuine myocardial disease. A recent study of the outcome of 8762 patients attending rapid access chest pain clinics showed that of 599 patients who reached the primary end-point (ie died from coronary heart disease or had a myocardial infarction or had a hospital admission with unstable angina) after a mean of 2.6 years, 194 (32 %) had non-cardiac chest pain. However, this represented only 2.7 % of the population (6396 people) with non-cardiac chest pain whereas 16.5 % of those with angina reached the primary end-point [1]. Those with identifiable disease therefore only accounted for a minority of these. There is also an additional group of patients who already have had clear cardiac disease that has apparently been treated successfully but who continue to have persistent chest pain that cannot be explained cardiologically. Together, these constitute a psychosomatic majority, who often suffer greatly from their symptoms and attend repeatedly with their symptoms, only to be reassured that they have no active cardiac disease. Approximately 75 % of these patients satisfy the diagnosis for a mental state disorder, mainly panic disorder, depressive and other anxiety disorders [2]. These problems are not usually recognised as requiring mental health interventions by either the patients or their doctors, and, because these problems tend to be persistent, they tend to become repeat attenders at rapid access chest pain or cardiology clinics, often get admitted to hospital for further checks and tests, and yet very few develop significant cardiac pathology [3].

Cognitive behaviour therapy and related psychological treatments are normally effective in the treatment of anxiety and depressive disorders but have not shown quite such success in the treatment of non-cardiac chest pain. Kisely et al [4, 5] have carried out Cochrane systematic reviews and found 8 randomised trials, increasing to 10 in their updated review, but the benefits were relatively modest. They concluded that these trials ‘suggested a modest to moderate benefit for psychological interventions, particularly those using a cognitive-behavioural framework, which was largely restricted to the first three months after the intervention. The evidence for brief interventions was less clear. Further RCTs of psychological interventions with follow-up periods of at least 12 months are said to be needed [4]. In their later review they added that hypnotherapy might be useful [5].

Examination of the reasons for the relatively poor performance of psychological treatments for this condition suggest three factors handicap progress; (i) the resistance of patients to the notion of a psychological explanation for their condition, (ii) the confidence of the therapists in dealing with a condition that mimics major cardiac pathology, and (iii) the ability of therapists to deliver treatment well to such patients. Most well-trained CBT therapists are psychologists but they do not have the background knowledge of medicine that helps to reassure the patients with non-cardiac chest pain that they understand their ‘medical’ problems; conversely, cardiac support nurses are excellent in this latter understanding but not so skilled in the former [6]. In the proposed study we have trained general nurses to a high standard (using formal fidelity checks with an approved scale) for this adapted form of cognitive behaviour therapy for non-cardiac chest pain and feel that both of the above requirements are now being met.

A recent pilot study in this population [7] has also demonstrated greatly reduced usage of accident and emergency service contact and in-patient bed usage after CBT, and so we judge that there is a strong possibility that the treatment will more than pay for itself in cost savings. We think we have such a treatment, and so are conducting a randomised controlled trial comparing adapted cognitive behaviour therapy for such patients, delivered by a trained team within the clinic, with standard support and reassurance in the clinic (standard treatment) and to compare outcomes in terms of symptoms, social functioning, hospital and emergency department attendances and admissions, over a period of 12 months. Full details will be obtained of all health service costs as it is predicted that, if the active treatment is successful, patients will attend hospital less often and have fewer investigations, and this will more than offset the cost of the psychological treatment.

## Methods/design

The design is a two-arm parallel design randomized controlled trial carried out in three centres that compares a modified form of cognitive behaviour therapy for chest pain (CBT-CP) that also includes elements of a similar successful treatment for health anxiety (CBT-HA [8] that has been found to have lasting benefit in reducing symptoms, as well as showing superiority for nurse-delivered treatment [9].

The study has two major research questions:

- does an adapted form of cognitive behaviour therapy for non-cardiac chest pain given between 2 to 6 sessions, lead to reduced anxiety over health over 6 months and one year?
- does this adapted form of cognitive behaviour therapy reduce health service costs over a period of one year?

These hypotheses, together with related secondary ones, are being tested in a two-arm parallel randomised controlled trial of 2–6 sessions of cognitive behaviour therapy for chest pain (CBT-CP) or standard treatment (ST) in patients presenting with non-cardiac chest pain to cardiology settings who have presented at least once before in the previous year and do not have cardiac pathology of sufficient severity to explain the symptoms.

Our previous work has suggested that most of these patients can be treated successfully in a relatively short number of sessions with an adapted form of cognitive behaviour therapy (CBT), although a minority of handicapped patients may need more. The randomisation was carried out by an independent Clinical Trials Unit (Health Services Unit, CHaRT, University of Aberdeen with equal allocation to CBT and ST, with initial help given from Open-CDMS, a similar independent unit. Standard treatment (ST) will consist of normal management at the clinics concerned, consisting of investigative procedures to exclude pathology and feedback to patients about the findings.

Assessments of outcome include reduction in health anxiety recorded with the Health Anxiety Inventory [10] (primary outcome at 6 months), and Lucock Health Anxiety Questionnaire [11] adapted with agreement of the author for chest pain, self-completed analog ratings of both the frequency and severity of chest pain and discomfort developed with the aid of a patient (Inskip Scale), self-ratings of generalised anxiety and depression (using the HADS scale) [12], social functioning using the Social Functioning Questionnaire (SFQ) [13], The Schedule for Evaluating Persistent Symptoms (SEPS) [14, 15], that has been found in preliminary studies to be an accurate measure of medically unexplained symptoms, and quality of life using the EQ-5D scale [16] six months and one year (all secondary outcomes). In addition, all health service related costs will be recorded in the 6 months before randomisation and at 6 month intervals subsequently until one year and the costs for patients in the two arms of the trial compared.

### Target number of participants

From previous work with the Health Anxiety Inventory we calculated that a difference between the scores of 4 points is a clinically significant difference (but this is currently being reassessed as 2 may be a more appropriate value). Using data from a similar randomised controlled trial [17] we demonstrated a significant benefit between CBT and control with a sample of 49 patients. In this study with a standard deviation for the change of HAI at 1 year as 6.0 a sample size of 96 patients would be have 90 % power to demonstrate significance at the two-sided 5 % significance level. However, it is expected that a current very similar project in New Zealand under the supervision of RM, with HT as an applicant, will provide very similar data from at least a subset of their sample that will allow some form of combination of data, such as individual meta-analysis, that will add to the power of the study.

#### Ethics and consent

All patients recruited to the study are initially referred by clinical staff involved in their care and are then seen by a research assistant who gives each person a participant information sheet and explains the study. If, after getting appropriate answers to questions, patients agree to take part in the randomised study, they sign a declaration of informed consent. The study has been approved by the NRES (Ethical) Committee East Midlands, Northampton, UK (11/EM/0376).

#### Procedure

The patients seen are those satisfying the criteria below who presented with chest pain to either cardiology clinics and/or accident and emergency departments at three hospitals, King's Mill Hospital, Sutton-in-Ashfield, Nottinghamshire, the Hillingdon Hospital, Middlesex and the Royal Berkshire Hospital, Reading. Two other centres were included but the lack of a local principal investigator prevented recruitment. The procedures for the chest pain pathway are not the same at these three hospitals but are likely to be representative of the UK as in most hospitals there is no standard pathway for the assessment of non-cardiac chest pain.

#### Inclusions and exclusion criteria

The criteria for inclusion are (a) significant chest pain on at least two separate occasions in the past year in which no significant pathology explaining the symptoms was found, (b) signed consent to take part in the study, (c) age between 18 and 75. The exclusion criteria are (a) under active psychiatric care, (b) having received a prescription of a new psychoactive drug within the previous two months, (c) receiving, or on waiting list for, a formal psychological treatment. Those who are currently stable and on regular psychoactive medication (for more than 2 months) are eligible for the study.

#### Randomisation

Patients who are identified as eligible for the trial by cardiology and accident and emergency staff, and willing to take part, were first assessed by an independent research assistant. After baseline assessment all ratings and demographic details are recorded on a secure on-line data base, initially Open-CDMS in London and from 2014, CHaRT in the Health Services Research Unit, University of Aberdeen. Patients are then allocated to either CBT-CP or standard care in permuted stacked blocks (do we specify numbers) stratified by study centre. The allocated treatment is then passed to the trial coordinator (SC) who, if the patient is allocated to CBT-CP, informs the next available therapist at the centre concerned and then the patient, GP and consultant. Patients allocated to standard care are informed by letter or phone call and the GP and hospital team also notified. Follow up assessments are carried out by research assistants ignorant of original allocation after 6 and 12 months. All data are kept by CHaRT until the termination of the trial.

#### Experimental interventions

Adapted CBT-CP (CBT for Chest Pain)

This will be given by staff trained and supervised by HT. This is similar in several ways to cognitive behaviour therapy for health anxiety (CBT-HA) [8] and its essential features are

- (i) A formulation made for a recent episode of chest pain with its central fear associations and possible consequences (by end of first session),
- (ii) assessment of the behaviours that are maintaining the chest pain,
- (iii) introducing the Beck equation of likelihood times awfulness divided by coping skills times rescue factors (a much larger issue for chest pain than for most other symptoms),
- (iv) building up a model of the cognitive theory of emotion with illustrations where necessary of the nature of symptoms,
- (v) introducing the model of fear of having heart disease/more serious heart disease (in those with pre-existing cardiac pathology) versus actually having heart disease/more serious heart disease,
- (vi) introducing the pie chart and pyramid systems for interpreting other worrying but innocuous symptoms,
- (vii) trying to influence the over-developed sense of responsibility that the patient feels is necessary to monitor the progress of, and likely interventions for, the chest pain,
- (viii) using the patient's strengths in finding alternative strategies of dealing with chest pain,
- (ix) developing a strategy for managing risk (highly important in this population),
- (x) diary homework to illustrate the relationship between symptoms and events likely to provoke anxiety.

In preliminary work we have found in some cases only one or two sessions of 50–60 min are needed to complete treatment but in others many more sessions are needed, particularly if the chest pain has become chronic.

#### Standard care

This constitutes the care that is currently given to patients in primary and secondary care at present; this involves appropriate testing, explanation of findings, reassurance of the implications of these and the opportunity for the patient to ask questions about the symptoms and the test results.

#### Economic analysis

The economic evaluation will take a health care perspective and, using the service use data collected over follow-up, will calculate the total cost of the services used by each study participant. These costs will be taken together with the cost of the CBT to generate average costs for each randomised group. These costs will be compared using standard t-tests, which are recommended for economic analysis because they allow for analysis of mean costs without transformation. The robustness of this approach will be checked through the calculation of bootstrapped confidence intervals [18, 19]. A cost-utility analysis will also be carried out where the costs in the CBT-CP and ST groups will be compared alongside the difference in QALYs, derived from the EQ-5D.

#### Primary and secondary outcomes

The primary outcome is the reduction in scores on the Health Anxiety Inventory between baseline and 6 months, Secondary outcomes are (i) the reduction in visual analog scores of frequency and intensity of chest pain, (ii) reduction in Lucock scores, (iii) reduction in the total SEPS score, (between baseline and 6 months and one year, (ii) the number of attendances at Accident & Emergency Departments after 6 months and one year, (iii) total health service costs in primary and secondary care at 6 and 12 months, (iv) reduction in generalised anxiety symptoms (on the HADS-Anxiety Scale after 6 months and 1 year, (v) reduction in depressive symptoms on the HADS-Depression Scale at the same time points, and (vi) change in mean Lucock scores from baseline after one year.

#### Analyses

All analyses will be by intention to treat, analysed as randomised. The primary analysis will compare mean change scores of the Health Anxiety Inventory scale from the baseline to 6 months between the treatment and control groups. We shall conduct sensitivity analyses to examine data missing mechanism to decide whether imputation approaches are necessary. Random effects regression model will be used to estimate and test differences of mean change scores between the two comparison groups at 6 and 12 months simultaneously with adjustment for baseline score and key variables used for minimisation procedure. We shall also examine distribution of the outcome score. If a skewed distribution such as a Poisson one was shown, data transformation such as logarithmic transformation of raw data will be carried out before any statistical analysis.

Analyses of secondary outcomes will follow the same analytic procedure and statistical approaches. Economic analysis will follow a similar methodology except that wherever possible data will be analysed without transformation and using bootstrapping techniques for skewed data [18, 19].

#### Fidelity of treatment

Each therapist will be trained by HT and assessed in vivo at interviews with patients using an adaptation of the Cognitive Therapy Rating Scale (CTRS), a necessary change for a population that largely regards its anxiety as an appropriate mood to monitor health [20]. Supervision in vivo will continue until at least a 75 % of maximum score is achieved.

### Status of the trial

**Fig. 1:**
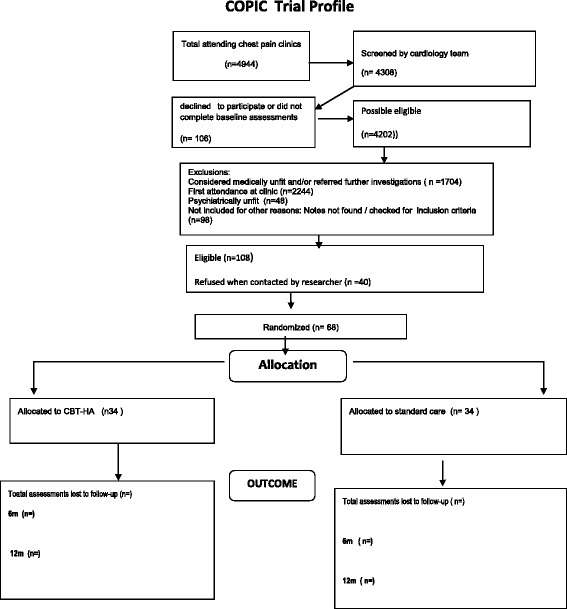
COPIC Trial Profile

Recruitment to the study began in July 2012 and the last patient was recruited in January 2015. No data have currently been examined. The CONSORT diagram illustrates the high numbers of people who present with potential non-cardiac chest pain (Fig. 1). 68 patients were recruited, a lower level than planned, but the revision in the clinically important difference (see above) makes the study only slightly underpowered.

## Discussion

The trial has been a difficult one to undertake because of the several ways in which non-cardiac chest pain is assessed in general. It has also highlighted a lack of close liaison between accident and emergency and cardiology departments, and surprise among many cardiologists that so many patients with non-cardiac chest pain are seen and discharged by accident and emergency staff without ever seeing a specialist in cardiology. There was also some evidence that dogmatic reluctance to follow any other route different from the standard investigation pathway sometimes hinders acceptance of a likely psychological explanation for symptoms. This is also reflected in the relatively high proportion of patients who refused randomisation (37 %) after passing through all other part of the assessment process. This reluctance to take part in psychological interventions has been noted by others in this field [6] and will probably only be overcome by increasing mental health literacy in both patients and hospital staff.
